# FEASIBILITY OF A DIGITAL ELDER MISTREATMENT INTERVENTION FOR COGNITIVELY IMPAIRED OLDER ADULTS

**DOI:** 10.1093/geroni/igad104.0478

**Published:** 2023-12-21

**Authors:** Fuad Abujarad, Chelsea Edwards, Judith Neugroschl, Ula Hwang, Richard Marottoli

**Affiliations:** Yale University School of Medicine, New Haven, Connecticut, United States; Yale University, New Haven, Connecticut, United States; Mount Sinai, New York City, New York, United States; Yale University, New Haven, Connecticut, United States; Yale Medical School, New Haven, Connecticut, United States

## Abstract

Older adults with cognitive impairments (CI) are at a higher risk for experiencing elder mistreatment (EM) and are less likely to report. Research is limited for self-administrated digital screening in this population, and generally focus on provider-based solutions to increasing EM identification. We developed a digital health intervention, VOICES, to increase EM identification. VOICES combines educational resources, screening, and brief psychoeducational interviewing to encourage older adults to self-report. We recruited participants with CI at a geriatric outpatient center (N=30) and the emergency department (ED) (N=101) to examine feasibility. Most participants could use VOICES independently (Montreal Cognitive Assessment (MoCA) scores ranging from 14-25). 87% of ED participants felt VOICES was appropriate for use in the ED, and 97% felt that the digital coach instructions were easy to understand. Our results suggest that the VOICES is feasible for EM screening in older adults with CI, specifically with MoCA scores ranging from 14-25.

